# Impact of statin therapy on coronary plaque composition: a systematic review and meta-analysis of virtual histology intravascular ultrasound studies

**DOI:** 10.1186/s12916-015-0459-4

**Published:** 2015-09-18

**Authors:** Maciej Banach, Corina Serban, Amirhossein Sahebkar, Dimitri P. Mikhailidis, Sorin Ursoniu, Kausik K. Ray, Jacek Rysz, Peter P. Toth, Paul Muntner, Svetlana Mosteoru, Hector M. García-García, G. Kees Hovingh, John JP Kastelein, Patrick W. Serruys, Lipid and Blood Pressure Meta-analysis Collaboration (LBPMC) Group

**Affiliations:** Department of Hypertension, Chair of Nephrology and Hypertension, Medical University of Lodz, Zeromskiego 113, 90-549 Lodz, Poland; Department of Functional Sciences, Discipline of Pathophysiology, Victor Babes University of Medicine and Pharmacy, Timisoara, Romania; Biotechnology Research Center, Mashhad University of Medical Sciences, Mashhad, Iran; Metabolic Research Centre, Royal Perth Hospital, School of Medicine and Pharmacology, University of Western Australia, Perth, Australia; Department of Clinical Biochemistry, Royal Free Campus, University College London Medical School, University College London, London, UK; Department of Functional Sciences, Discipline of Public Health, Victor Babes University of Medicine and Pharmacy, Timisoara, Romania; School of Public Health, Imperial College London, London, UK; Chair of Nephrology and Hypertension, Medical University of Lodz, Lodz, Poland; Preventive Cardiology, CGH Medical Center, Sterling, IL USA; The Johns Hopkins Ciccarone Center for the Prevention of Heart Disease, Baltimore, MD USA; Department of Epidemiology, University of Alabama at Birmingham, Birmingham, AL USA; Institute for Cardiovascular Medicine Timisoara, Cardiology Department, Victor Babes University of Medicine and Pharmacy, Timisoara, Romania; Department of Interventional Cardiology, Thoraxcenter, Erasmus Medical Centre, Rotterdam, The Netherlands; Cardialysis BV, Rotterdam, The Netherlands; Department of Vascular Medicine, Academic Medical Center, University of Amsterdam, Amsterdam, The Netherlands; Imperial College, London, UK

**Keywords:** Virtual histology intravascular ultrasound, VH-IVUS, Statins, Statin therapy

## Abstract

**Background:**

Virtual histology intravascular ultrasound (VH-IVUS) imaging is an innovative tool for the morphological evaluation of coronary atherosclerosis. Evidence for the effects of statin therapy on VH-IVUS parameters have been inconclusive. Consequently, we performed a systematic review and meta-analysis to investigate the impact of statin therapy on plaque volume and its composition using VH-IVUS.

**Methods:**

The search included PubMed, Cochrane Library, Scopus and Embase (through 30 November 2014) to identify prospective studies investigating the effects of statin therapy on plaque volume and its composition using VH-IVUS.

**Results:**

We identified nine studies with 16 statin treatment arms and 830 participants. There was a significant effect of statin therapy in reducing plaque volume (standardized mean difference (SMD): −0.137, 95 % confidence interval (CI): −0.255, −0.019; *P* = 0.023), external elastic membrane volume (SMD: −0.097, 95 % CI: −0.183, −0.011; *P* = 0.027) but not lumen volume (SMD: −0.025, 95 % CI: −0.110, +0.061; *P* = 0.574). There was a significant reduction in fibrous plaque volume (SMD: −0.129, 95 % CI: −0.255, −0.003; *P* = 0.045) and an increase of dense calcium volume (SMD: +0.229, 95 % CI: +0.008, +0.450; *P* = 0.043), while changes in fibro-fatty (SMD: −0.247, 95 % CI: −0.592, +0.098; *P* = 0.16) and necrotic core (SMD: +0.011, 95 % CI: −0.144, +0.165; *P* = 0.892) tissue volumes were not statistically significant.

**Conclusions:**

This meta-analysis indicates a significant effect of statin therapy on plaque and external elastic membrane volumes and fibrous and dense calcium volumes. There was no effect on lumen volume, fibro-fatty and necrotic tissue volumes.

## Background

Despite continuously improving therapies used for acute coronary syndromes (ACS), cardiovascular disease (CVD) and its complications remain the leading causes of mortality and morbidity [1]. The most important mechanism leading to ACS is the rupture of a vulnerable plaque and subsequent thrombus formation [2–4]. The lesion most frequently prone to rupture is represented by the thin-cap fibroatheroma (TCFA), which contains a large necrotic core with an overlying thin fibrous cap [5]. The recently introduced technique of virtual histology intravascular ultrasound (VH-IVUS) utilizes spectral analysis of the radiofrequency ultrasound backscatter signals, which allows *in vivo* differentiation of four distinct atherosclerotic plaque phenotypes: fibrous; fibro-fatty; dense calcium; and necrotic core [6]. *In vivo* studies of coronary [7] and carotid plaques [8] have demonstrated the accuracy of VH-IVUS for histological characterization of atherosclerotic plaques.

The Providing Regional Observations to Study Predictors of Events in the Coronary Tree (PROSPECT), the VH-IVUS in Vulnerable Atherosclerosis (VIVA) and the European Collaborative Project on Inflammation and Vascular Wall Remodeling in Atherosclerosis (ATHEROREMO-IVUS) substudy are three important prospective studies that have demonstrated that the presence of VH-IVUS-derived TCFA lesions is strongly and independently predictive for the occurrence of major adverse cardiovascular events (MACE) [9–11]. Extensive research has focused on preventing CVD events, including therapies that may stabilize atherosclerotic plaques [12]. There is a well-established association between therapy with high doses of statins and regression of coronary atherosclerosis [13]. Also, there have been studies that have investigated the efficiency of statin therapy on coronary plaque composition evaluated with the VH-IVUS method [14, 15]. However, these studies were conducted in relatively small study cohorts and are not conclusive. It is not established whether and to what extent statins have an effect on coronary plaque composition. The purpose of this meta-analysis was therefore to investigate the impact of statin therapy on coronary plaque composition.

## Methods

### Data sources

This study was designed according to the guidelines of the 2009 Preferred Reporting Items for Systematic Reviews and Meta-Analysis (PRISMA) statement [16]. Our search included Scopus, Medline, Web of Science and Cochrane Library databases. It was limited to prospective studies carried out up to 30 November 2014, investigating the potential effects of statin therapy on plaque volume and its composition. The databases were searched using the following search terms in titles and abstracts (also in combination with Medical Subject Headings (MeSH) terms): ‘virtual histology intravascular ultrasound’ OR ‘virtual histology IVUS’ OR ‘VH IVUS’ OR ‘VH-IVUS’ AND ‘statins’ (all fields) OR ‘statin’ (all fields) OR ‘statin therapy’ (all fields) OR ‘rosuvastatin’ OR ‘pravastatin’ OR ‘fluvastatin’ OR ‘simvastatin’ OR ‘atorvastatin’ OR ‘pitavastatin’ OR ‘lovastatin’ OR ‘cerivastatin’ AND ‘virtual histology intravascular ultrasound’ (all fields) OR ‘virtual histology IVUS’ (all fields) OR ‘VH IVUS’ (all fields) OR ‘VH-IVUS’ (all fields). The wild-card term ‘*’ was used to increase the sensitivity of the search strategy. No language restriction was used in the literature search. The search was limited to studies in humans. References of all obtained articles were additionally explored for supplemental publications. Two reviewers (CS and AS) examined every article separately to minimize the possibility of duplication, investigating reviews, case studies and experimental studies. Disagreements were managed by discussion with a third party (MB).

### Study selection

#### Inclusion criteria

Original studies were included if they met the following inclusion criteria: a) being a prospective clinical study; b) investigating the impact of statin therapy on plaque volume and/or its composition using VH-IVUS (in comparison to placebo group or high-intensity versus moderate/low-intensity statin therapy); c) presentation of sufficient information on VH-IVUS findings at baseline and at the end of study; and d) statin therapy for at least 2 weeks.

#### Exclusion criteria

Exclusion criteria were: a) non-clinical studies (experimental and basic studies); b) observational or retrospective studies; c) duplicate reports or secondary or *post hoc* analyses of the same study population; and d) lack of sufficient information on baseline or follow-up VH-IVUS data. Exclusion of an article for this reason was also done if no feedback was received after contacting the author(s).

#### Data extraction

Eligible studies were reviewed and the following data were abstracted: 1) first author’s name; 2) year of publication; 3) study location; 4) number of participants; 5) age, gender and body mass index (BMI) of study participants; 6) baseline levels of total cholesterol (TC), low-density lipoprotein cholesterol (LDL-C), high-density lipoprotein cholesterol (HDL-C), triglycerides (TG), high-sensitivity C-reactive protein (hs-CRP) and glucose; 7) systolic (SBP) and diastolic blood pressure (DBP); 8) statin type, statin dose and duration of treatment (both in research and control groups); and 9) data regarding baseline and follow-up VH-IVUS findings including plaque volume (PV), lumen volume (LV), external elastic membrane volume (EEMV), as well as atheroma compositional data (comprising volumes of fibrous, fibro-fatty, dense calcium and necrotic core tissues).

#### Quality assessment and quantitative data synthesis

The quality of included studies was assessed using the Cochrane scale. Meta-analysis was conducted using Review Manager, version 5.2 (Cochrane Collaboration, Oxford, UK), and Comprehensive Meta-Analysis (CMA) V2 software (Biostat, NJ, USA) [17]. Standard deviations (SD) of the mean difference were calculated using the following formula: SD = square root ((SD_pre-treatment_)^2^ + (SD_post-treatment_)^2^ − (2R × SD_pre-treatment_ × SD_post-treatment_)), assuming a correlation coefficient (R) = 0.5. In case of reporting SEM, SD was estimated using the following formula: SD = SEM × sqrt (*n*), where *n* is the number of subjects. In case levels were reported as the median and interquartile range, the mean and SD were estimated using the recommendations of Hozo et al. [18].

Net changes in measurements (change scores) were calculated for parallel and crossover trials, as follows: measure at end of follow-up − measure at baseline. A random-effects model (using DerSimonian–Laird method) and the generic inverse variance method were used to compensate for the heterogeneity of studies in terms of statin type, statin dose, study design, treatment duration and the characteristics of populations being studied [19]. Effect sizes were expressed as weighed standardized mean difference (SMD) and 95 % confidence intervals (CI). In order to evaluate the influence of each study on the overall effect size, sensitivity analysis was conducted using the one-study remove (leave-one-out) approach.

#### Meta-regression

Meta-regression was performed using a random-effects model (using unrestricted maximum likelihood method) to evaluate the association between calculated SMD in plaque volume with duration of statin therapy and changes in LDL-C concentrations.

#### Publication bias

Potential publication bias was explored using visual inspection of Begg’s funnel plot asymmetry, and Begg’s rank correlation and Egger’s weighted regression tests. The Duval and Tweedie ‘trim and fill’ and ‘fail-safe N’ methods were used to adjust the analysis for the effects of publication bias [20].

## Results

### Search results and trial flow

**Fig. 1:**
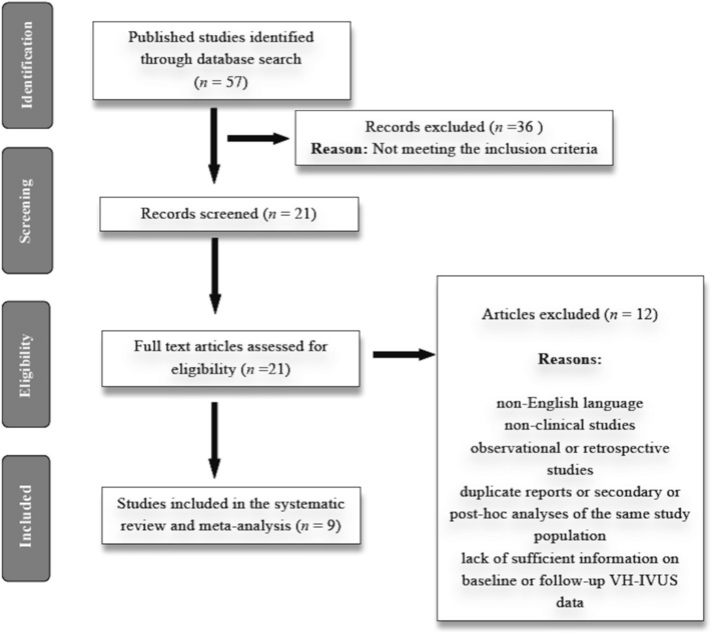
Flow diagram for study selection. VH-IVUS, virtual histology intravascular ultrasound

A total of nine eligible studies comprising 16 treatment arms met the inclusion criteria and were included for the final meta-analysis [14, 21–28]. An overview of the study selection process is presented in Fig. 1.

### Characteristics of included studies

Among 830 participants in the included studies, 737 were allocated to statin intervention groups (with different statin preparations and different doses) and 93 to placebo group. The number of participants in these studies ranged from 20 to 228. The studies were published between 2009 and 2014, and were conducted in USA (two studies), South Korea (two studies), China, Hong Kong and Japan (three studies). The following statin doses were administered in the included trials: 10 to 80 mg/day atorvastatin; 10 to 40 mg/day pravastatin; 20 mg/day simvastatin; 10 to 40 mg/day rosuvastatin; 60 mg/day fluvastatin; and 2 to 4 mg/day pitavastatin. One study did not mention statin preparation or dosage [24]. Duration of statin intervention ranged from 6 to 24 months. Only two studies were placebo-controlled, the other seven included only statin intervention groups. Demographic and baseline parameters of the included studies are shown in Table 1.

**Table 1:** Demographic characteristics of the included studies

Study		Eshtehardi et al. [21]	Guo et al. [22]	Hong et al. [23]	Hwang et al. [24]	Lee et al. [14]	Nasu et al. [25]	Nozue et al. [26]	Puri et al. [27]	Taguchi et al. [28]
Year		2012	2012	2009	2013	2012	2009	2012	2014	2013
Location		USA	China	Korea	Korea	Hong Kong	Japan	Japan	USA	Japan
Design		Pilot study on consecutive patients treated with atorvastatin	Randomized placebo-controlled parallel group trial	Randomized parallel group trial	Prospective study on patients treated with statin	Prospective randomized double-blind parallel group trial	Prospective and multicenter study with non-randomized and no blinded design	Prospective, open-labeled, randomized, multicenter study	Randomized parallel-group trial	Prospective, non-randomized, non-controlled and open-label trial
Duration of study		6 months	6 months	12 months	6 months	6 months	12 months	8 months	24 months	8–10 months
Inclusion criteria		Patients with an abnormal non-invasive stress test, stable angina or stabilized acute coronary syndrome who were found to have moderate lesions requiring invasive physiologic evaluation	Coronary heart disease patients with stable atherosclerotic plaques	Patients with *de novo* non-culprit/non-target lesions without significant stenosis by coronary angiogram (diameter stenosis <50 %), lesions with a plaque burden <0.75 by gray-scale IVUS, and lesions located in 1 of 3 major epicardial arteries in which stent implantation was not performed	Patients with acute coronary syndrome	Statin-naive patients free from unstable angina >8 weeks before intervention or acute coronary syndrome and with angiographic critical coronary stenosis requiring percutaneous coronary intervention	Patients older than 30 years of age with symptomatic stable angina pectoris. Angiographic inclusion criteria: 1) target vessel for VH-IVUS interrogation must not have undergone angioplasty or have more than 50 % luminal narrowing throughout a target segment with a minimum length of 30 mm; 2) target vessel for VH-IVUS interrogation had mild-to-moderate vessel tortuosity and calcification for safe and accurate examination; and 3) left ventricular ejection fraction >30 %	Patients with stable and unstable angina after successful percutaneous coronary intervention	Patients with angiographically demonstrable coronary disease and LDL-C <116 mg/dL, following a 2-week treatment period with atorvastatin (40 mg) or rosuvastatin (20 mg) daily	Patients with acute coronary syndrome defined as unstable angina of Braunwald class IIIB (angina at rest without increased levels of the creatine kinase-MB fraction within 24 hours before coronary angiography), non-ST-segment elevation myocardial infarction, or ST-segment elevation myocardial infarction
Statin form		Atorvastatin	Atorvastatin	Simvastatin or rosuvastatin	NS	Atorvastatin	Fluvastatin	Pitavastatin or pravastatin	Rosuvastatin or atorvastatin	Atorvastatin or pitavastatin
Statin intervention		80 mg/day	10–80 mg/day	20 mg/day or 10 mg/day	NS	10–40 mg/day	60 mg/day	4 mg/day or 20 mg/day	40 mg/day or 80 mg/day	10 mg/day or 2 mg/day
Participants	Intervention	20	47^a^	50^e^	54	19^a^	40	58^g^	36^i^	60^a^
			45^b^							
			43^c^	50^f^		20^c^		61^h^	35^d^	60^j^
			39^d^							
	Control	-	54	-	-	-	39		-	-
Age (years)	Intervention	54 (46–68)	62.64 ± 12.0^a^	58 ± 10^e^	59 ± 10	65.05 ± 9.99^a^	63 ± 10	66 ± 9^g^	57.6 ± 9.0**	65.8 ± 16.2^#^
			59.18 ± 8.48^b^							
			58.91 ± 12.90^c^	59 ± 9^f^		63.70 ± 9.80^c^		67 ± 11^h^		63.7 ± 16.5^##^
			58.95 ± 9.68^d^							
	Control	-	62.07 ± 8.51	-	-	-	62 ± 12	-	-	-
Male (%)	Intervention	65.0	88.88^a^	80.0^e^	70.37	73.68^a^	80.0	89.65^g^	80.3**	76.6^#^
			85.10^b^							
			80.0^c^	74.0^f^		90.0^c^		77.05^h^		69.2^##^
			95.35^d^							
	Control	-	87.18	-	-	-	77.5	-	-	-
BMI (kg/m^2^)	Intervention	30 (27–36)	NS^a^	NS^e^	NS	26.83 ± 6.85^a^	NS	24.4 ± 3.5^g^	28.6 ± 4.5**	24.0 ± 2.5^#^
			NS^b^							
			NS^c^	NS^f^		26.58 ± 5.44^c^		24.5 ± 3.3^h^		24.2 ± 2.7^##^
			NS^d^							
	Control	-	NS	-	-	-	NS	-	-	-
hs-CRP (mg/L)	Intervention	NS	6.04 ± 2.52^a^	0.17 ± 0.22^e^	3.18 ± 5.29	NS^a^	2.05 ± 2.20	3.76 (1.22–9.22)^g^	1.4 (0.7–2.7)**	NS^#^
			5.09 ± 1.94^b^							
			5.67 ± 2.22^c^	0.21 ± 0.20^f^		NS^c^		4.23 (1.21–9.26)^h^		NS^##^
			6.10 ± 2.12^d^							
	Control	-	5.07 ± 1.80	-	-	-	1.19 ± 1.03	-	-	-
Total cholesterol (mg/dL)	Intervention	186.0 (168.0–212.5)	NS^a^	191 ± 34^e^	195.0 ± 35.9	200.58 ± 41.54^a^	239.1 ± 32.8	199 ± 34^g^	203.1 ± 38**	NS^#^
			NS^b^							
			NS^c^	189 ± 27^f^		184.17 ± 29.27^c^		210 ± 38^h^		NS^##^
			NS^d^							
	Control	-	NS	-	-	-	199.5 ± 22.8	-	-	-
LDL-C (mg/dL)	Intervention	118.5 (105.3–140.5)	116.96 ± 27.02^a^	119 ± 30^e^	119.7 ± 31.4	122.39 ± 39.54^a^	144.9 ± 31.5	126 ± 28^g^	128.6 ± 30.7**	117.3 ± 34.7^#^
			112.71 ± 23.93^b^							
			111.94 ± 13.12^c^	116 ± 28^f^		112.35 ± 27.14^c^		137 ± 35^h^		116.2 ± 26.7^##^
			109.24 ± 25.48^d^							
	Control	-	113.48 ± 27.79	-	-	-	122.3 ± 18.9	-	-	-
HDL-C (mg/dL)	Intervention	39.5 (33.3–52.8)	34.74 ± 6.56^a^	43 ± 10^e^	38.9 ± 8.5	41.47 ± 9.46^a^	52.7 ± 12.4	46 ± 11^g^	44.7 ± 11.0**	46.8 ± 10.9^#^
			35.90 ± 7.72^b^							
			37.44 ± 9.26^c^	43 ± 11^f^		42.82 ± 17.45^c^		47 ± 11^h^		46.5 ± 11.4^##^
			34.74 ± 5.02^d^							
	Control	-	37.06 ± 6.95	-	-	-	54.3 ± 17.8	-	-	-
Triglycerides (mg/dL)	Intervention	115.5 (83.5–158.8)	NS^a^	149 ± 69^e^	178.5 ± 126.1	168.58 ± 96.19^a^	200.6 ± 125.4	129 ± 73^g^	130 (99–191)**	115.6 ± 22.6^#^
			NS^b^							
			NS^c^	152 ± 75^f^		154.42 ± 1.02^c^		134 ± 58^h^		119.9 ± 35.2^##^
			NS^d^							
	Control	-	NS	-	-	-	122.8 ± 50.1	-	-	
Glucose (mg/dL)	Intervention	NS	103.14 ± 18.0^a^	NS^e^	NS	NS^a^	NS	NS^g^	NS**	NS^#^
			102.96 ± 14.76^b^							
			90.0 ± 14.94^c^	NS^f^		NS^c^		NS^h^		NS^##^
			101.34 ± 17.46^d^							
	Control	-	94.68 ± 17.64	-	NS	-	NS	-	-	-
SBP (mmHg)	Intervention	129 (114–145)	NS^a^	NS^e^	NS	NS^a^	NS	NS^g^	NS**	NS^#^
			NS^b^							
			NS^c^	NS^f^		NS^c^		NS^h^		NS^##^
			NS^d^							
	Control	-	NS	-	-	-	NS	-	-	-
DBP (mmHg)	Intervention	72 (68–83)	NS^a^	NS^e^	NS	NS^a^	NS	NS^g^	NS**	NS^#^
			NS^b^							
			NS^c^	NS^f^		NS^c^		NS^h^		NS^##^
			NS^d^							
	Control	-	NS	-	-	-	NS	-	-	-
Plaque volume (mm^3^)	Intervention	308.8 (236.8–432.6)	38.07 ± 13.94^a^	88.3 ± 26.9^e^	76.1 ± 32.1	98.47 ± 70.84^a^	440.2 ± 220.3	9.06 ± 2.90^g^*	146.0 ± 55.6**	10.2 ± 3.0^#^*
			33.83 ± 10.56^b^							
			37.06 ± 12.01^c^	91.5 ± 27.5^f^		144.17 ± 154.46^c^		8.83 ± 3.67^h^*		9.9 ± 2.9^##^*
			36.47 ± 14.68^d^							
	Control	-	34.83 ± 13.76	-	-	-	432.9 ± 247.5	-	-	-
Lumen volume (mm^3^)	Intervention	427.3 (310.9–703.7)	NS^a^	85.2 ± 20.4^e^	70.5 ± 24.1	NS^a^	373.7 ± 188.4	7.40 ± 2.55^g^*	214.9 ± 71.5**	6.6^#^*^§^
			NS^b^							
			NS^c^	87.6 ± 26.2^f^		NS^c^		7.42 ± 2.66^h^*		8.0 ± 2.8^##^*
			NS^d^							
	Control	-	NS	-	-	-	444.7 ± 233.5	-	-	-
External elastic membrane volume (mm^3^)	Intervention	830.9 (606.8–1,080.1)	NS^a^	173.5 ± 37.1^e^	146.6 ± 52.3	NS^a^	813.9 ± 398.5	16.46 ± 4.98^g^*	360.9 ± 108.8**	16.8 ± 4.6^#^*
			NS^b^							
			NS^c^	179.1 ± 46.6^f^		NS^c^		16.25 ± 5.63^h^*		17.9 ± 5.0^##^*
			NS^d^							
	Control	-	NS	-	-	-	877.6 ± 458.3	-	-	-
Fibrous volume (mm^3^)	Intervention	89.9 (67.1–123.9)	NS^a^	25.6 ± 12.7^e^	27.7 ± 15.6	37.04 ± 30.41^a^	146.5 ± 85.6	3.46 ± 1.65^g^*	18.5 (9.8–29.3)**	5.9 ± 2.6^#^*
			NS^b^							
			NS^c^	28.2 ± 14.4^f^		54.90 ± 58.05^c^		3.13 ± 1.98^h^*		5.8 ± 2.3^##^*
			NS^d^							
	Control	-	NS	-	-	-	142.9 ± 113.3	-	-	-
Fibro-fatty volume (mm^3^)	Intervention	10.6 (6.4–27.9)	NS^a^	4.1 ± 2.9^e^	4.5 ± 3.9	9.76 ± 9.80^a^	80.1 ± 57.9	1.09 ± 0.88^g^*	23.1 (8.8–36.3)**	1.5 ± 1.1^#^*
			NS^b^							
			NS^c^	4.5 ± 4.0^f^		19.39 ± 36.04^c^		1.05 ± 1.03^h^*		0.7 ± 0.6^##^*
			NS^d^							
	Control	-	NS	-	-	-	50.7 ± 32.9	-	-	-
Dense calcium volume (mm^3^)	Intervention	10.5 (4.0–20.9)	NS^a^	6.5 ± 6.3^e^	4.2 ± 3.2	3.18 ± 3.44^a^	9.4 ± 9.9	0.42 ± 0.35^g^*	1.2 (0.2–3.8)**	0.6^#^*^§^
			NS^b^							
			NS^c^	6.8 ± 6.4^f^		4.85 ± 7.68^c^		0.44 ± 0.47^h^*		0.6^##^*^§^
			NS^d^							
	Control	-	NS	-	-	-	13.7 ± 12.7	-	-	-
Necrotic core volume (mm^3^)	Intervention	30.8 (13.9–48.2)	NS^a^	15.8 ± 11.3^e^	8.7 ± 6.4	7.91 ± 7.47^a^	21.4 ± 24.9	0.68 ± 0.42^g^*	5.9 (2.6–12.3)**	1.6 ± 0.9^#^*
			NS^b^							
			NS^c^	15.5 ± 8.4^f^		11.89 ± 18.72^c^		0.80 ± 0.66^h^*		2.1 ± 1.4^##^*
			NS^d^							
	Control	-	NS	-	-	-	22.1 ± 17.4	-	-	-

Values are expressed as mean ± SD or median (25–75 percentiles). ^a^10 mg/day atorvastatin arm; ^b^20 mg/day atorvastatin arm; ^c^40 mg/day atorvastatin arm; ^d^80 mg/day atorvastatin arm; ^e^20 mg/day simvastatin arm; ^f^10 mg/day rosuvastatin arm; ^g^4 mg/day pitavastatin arm; ^h^20 mg/day pravastatin arm; ^i^40 mg/day rosuvastatin arm; ^j^2 mg/day pitavastatin arm; ^*^the value was provided as volume index defined as the volume divided by the segment length (mm^3^/mm); ^**^the value was provided for rosuvastatin and atorvastatin arms together; ^#^patients belonging to plaque regression group (n = 94); ^##^patients belonging to plaque progression (n = 26) group; ^§^SD not shown. BMI, body mass index; DBP, diastolic blood pressure; HDL-C, high-density lipoprotein cholesterol; hs-CRP, high-sensitivity C-reactive protein; IVUS, intravascular ultrasound; LDL-C, low-density lipoprotein cholesterol; MB, myocardial band; NS, not stated; SBP, systolic blood pressure; VH-IVUS, virtual histology intravascular ultrasound

### Risk of bias assessment

According to the Cochrane Collaboration [29], a specific tool for assessing risk of bias in every study involved consists of selection of particular characteristics of the study. This involves assessing the risk of bias as ‘low risk’, ‘high risk or ‘unclear risk’. The last category reveals either lack of detail or concern over the potential for bias. There are seven examined fields including: sequence generation (selection bias); allocation sequence concealment (selection bias); blinding of participants and personnel (performance bias); blinding of outcome assessment (detection bias); incomplete outcome data (attrition bias); selective outcome reporting (reporting bias); and other potential sources of bias (Table 2).

**Table 2:** Assessment of risk of bias in the included studies using Cochrane criteria

Study	Reference	Sequence generation	Allocation concealment	Blinding of participants and personnel	Blinding of outcome assessment	Incomplete outcome data	Selective outcome reporting	Other potential threats to validity
Eshtehardi et al. 2012	[21]	H	H	H	H	L	L	L
Guo et al. 2012	[22]	U	U	H	H	L	L	L
Hong et al. 2009	[23]	U	U	H	H	L	L	L
Hwang et al. 2013	[24]	H	H	H	L	L	L	H
Lee et al. 2012	[14]	L	L	L	L	L	L	L
Nasu et al. 2009	[25]	H	H	H	H	L	L	L
Nozue et al. 2012	[26]	L	L	H	L	L	L	L
Puri et al. 2014	[27]	U	U	H	H	L	L	L
Taguchi et al. 2013	[28]	H	H	H	H	L	L	L

H, high risk of bias; L, low risk of bias; U, unclear risk of bias

### Quantitative data synthesis

**Fig. 2:**
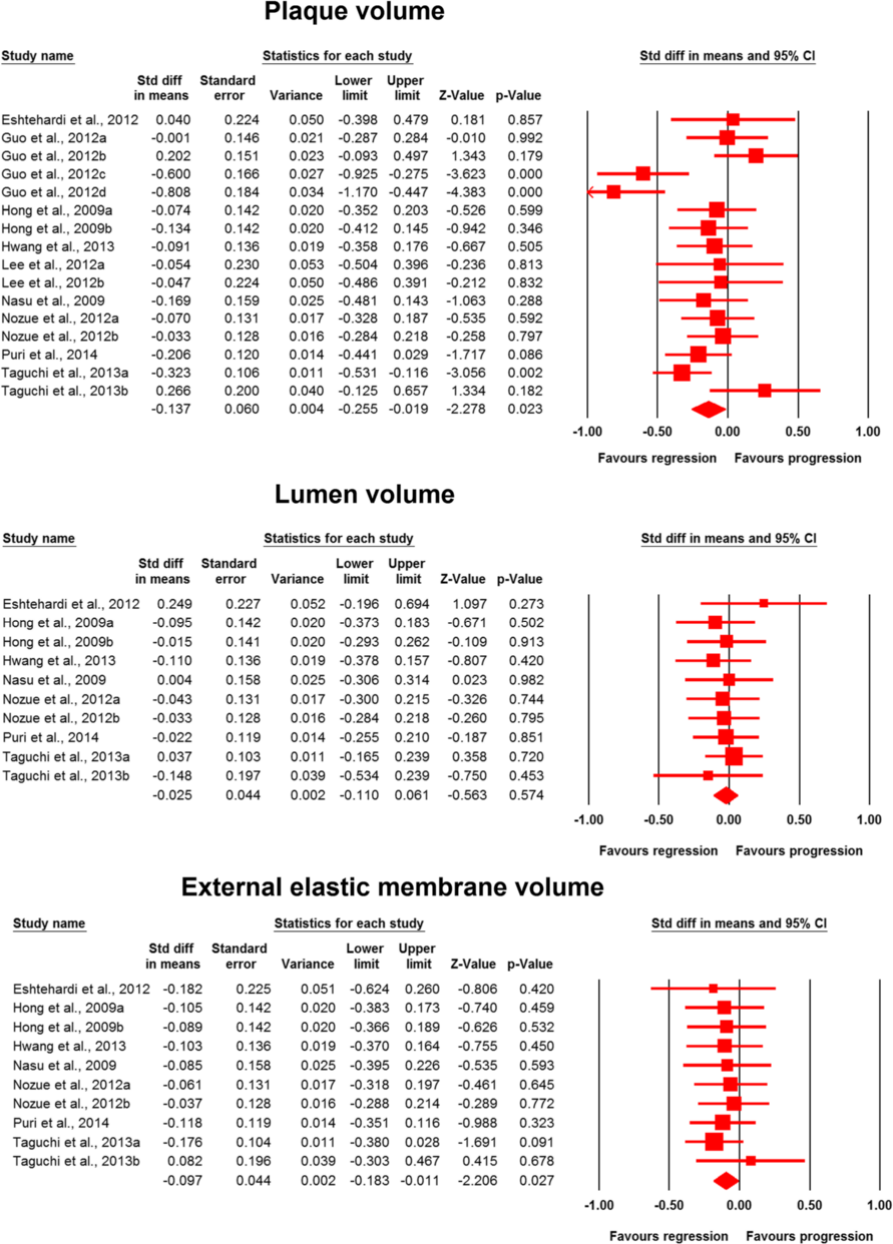
Forest plot detailing weighted mean difference and 95 % confidence intervals for the impact of statin therapy on plaque, lumen and external elastic membrane volumes according to virtual histology intravascular ultrasound (VH-IVUS). Meta-analysis was performed using a random-effects model with inverse variance weighting

**Fig. 3:**
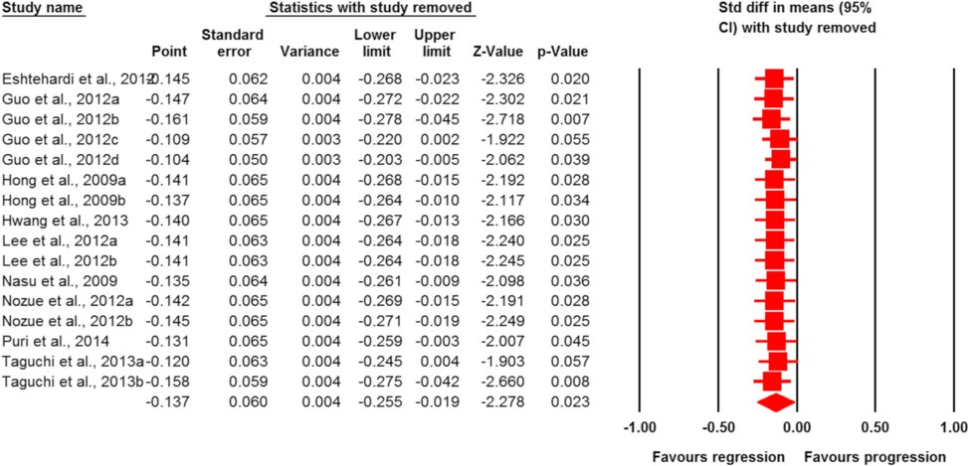
Leave-one-out sensitivity analysis of the impact of statin therapy on plaque volume

Meta-analysis of data from 16 statin-treated arms showed a significant effect of statin therapy in reducing plaque volume (SMD: −0.137, 95 % CI: −0.255, −0.019; *P* = 0.023) (Fig. 2). This effect size was robust in the sensitivity analysis and remained at a significant or borderline significant levels following omission of each single study (Fig. 3). Statin therapy was also associated with a significant decrease in EEMV (SMD: −0.097, 95 % CI: −0.183, −0.011; *P* = 0.027) but not LV (SMD: −0.025, 95 % CI: −0.110, +0.061; *P* = 0.574) (Fig. 2).

**Fig. 4:**
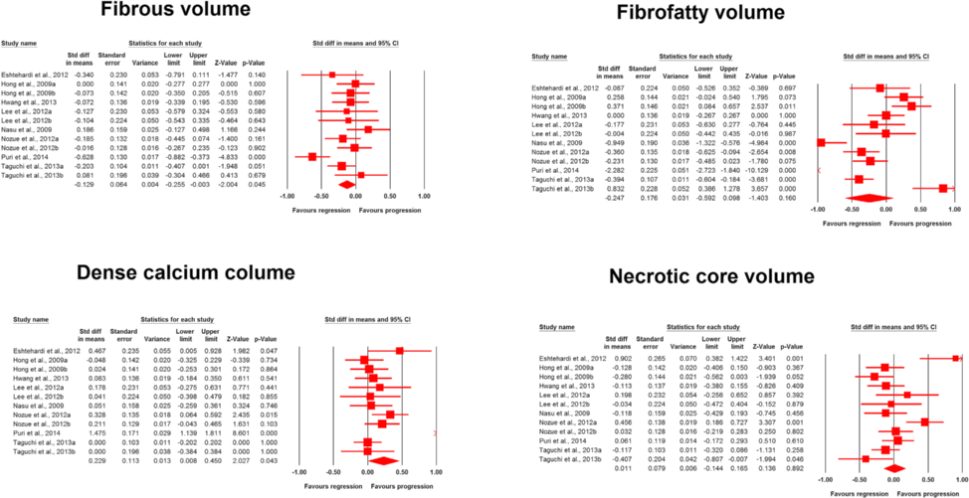
Forest plot detailing weighted mean difference and 95 % confidence intervals for the impact of statin therapy on plaque composition parameters according to virtual histology intravascular ultrasound (VH-IVUS). Meta-analysis was performed using a random-effects model with inverse variance weighting

The analysis of plaque composition data indicated significant reduction in fibrous (SMD: −0.129, 95 % CI: −0.255, −0.003; *P* = 0.045) and increase in dense calcium (SMD: 0.229, 95 % CI: 0.008, 0.450; *P* = 0.043) volumes, while fibro-fatty (SMD: −0.247, 95 % CI: −0.592, +0.098; *P* = 0.160) and necrotic core (SMD: 0.011, 95 % CI: −0.144, +0.165; *P* = 0.892) tissue volumes remained statistically unaltered (Fig. 4).

**Fig. 5:**
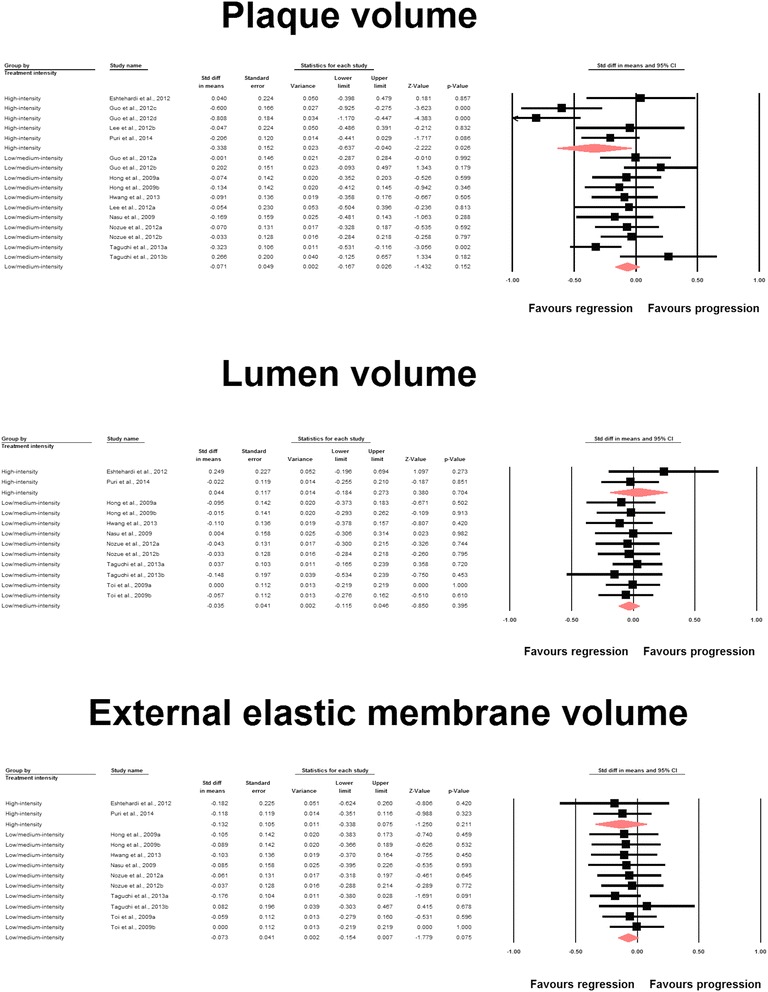
Forest plot detailing weighted mean difference and 95 % confidence intervals for the impact of high-intensity versus moderate/low-intensity statin therapy on plaque, lumen and external elastic membrane volumes according to virtual histology intravascular ultrasound (VH-IVUS). Meta-analysis was performed using a random-effects model with inverse variance weighting

**Fig. 6:**
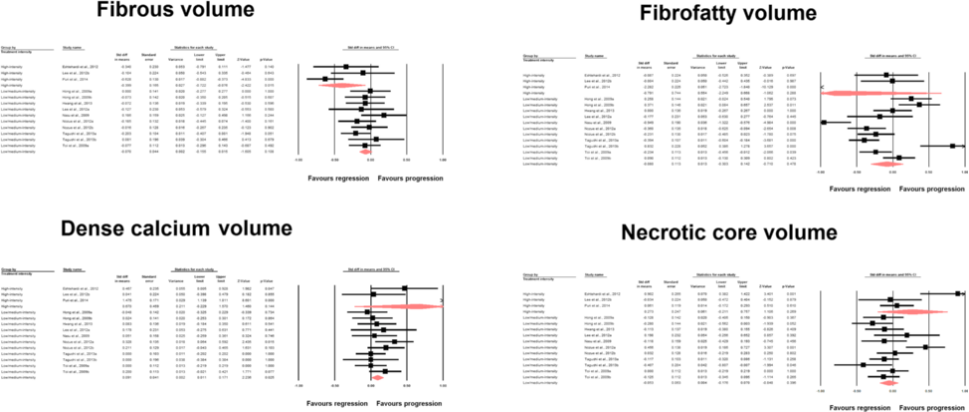
Forest plot detailing weighted mean difference and 95 % confidence intervals for the impact of high-intensity versus moderate/low-intensity statin therapy on plaque composition parameters according to virtual histology intravascular ultrasound (VH-IVUS). Meta-analysis was performed using a random-effects model with inverse variance weighting

A subgroup analysis was performed to compare the impact of high-intensity versus moderate/low-intensity statin therapy on coronary atherosclerosis according to American College of Cardiology (ACC)/American Heart Association (AHA) lipid guidelines [30]. High-intensity statin therapy had a greater effect in reducing plaque volume (SMD: −0.338, 95 % CI: −0.637, −0.040; *P* = 0.026) compared with moderate/low-intensity treatment (SMD: −0.071, 95 % CI: −0.167, +0.026; *P* = 0.152) (Fig. 5). However, no significant difference between the subgroups was observed in terms of effects on LV and EEMV (Fig. 5). With respect to plaque composition parameters, significant changes in dense calcium (SMD: 0.091, 95 % CI: 0.011, 0.171; *P* = 0.025) and fibrous (SMD: −0.399, 95 % CI: −0.722, −0.076; *P* = 0.015) volumes were observed in the moderate/low-intensity and high-intensity subgroups, respectively (Fig. 6). The effects of both treatment regimens on fibro-fatty and necrotic core tissue volumes were statistically comparable (Fig. 6).

Another subgroup analysis was performed to compare the effects of statin therapy on coronary atherosclerosis in the subgroups of trials with and without ACS patients. PV was reduced only in the subset of trials not recruiting ACS patients (SMD: −0.175, 95 % CI: −0.334, −0.015; *P* = 0.032). The impact of statin therapy on other indices in ACS+ and ACS− subgroups are summarized in Table 3.

**Table 3:** Comparison of the effects of statin therapy on coronary atherosclerosis indices in subgroups of trials recruiting subjects with and without ACS

	Without ACS			With ACS		
	SMD	95 % CI	*P* value	SMD	95 % CI	*P* value
Plaque volume	−0.175	−0.334, −0.015	0.032	−0.080	−0.258, 0.099	0.382
Lumen volume	−0.033	−0.121, 0.056	0.469	−0.007	−0.148, 0.134	0.919
External elastic membrane volume (mm^3^)	−0.065	−0.154, 0.024	0.150	−0.121	−0.263, 0.020	0.093
Fibrous volume (mm^3^)	−0.010	−0.053, 0.133	0.888	0.027	−0.243, 0.297	0.844
Fibro-fatty volume	−0.395	−0.824, 0.034	0.071	0.008	−0.312, 0.328	0.961
Dense calcium volume	−0.119	−0.304, 0.065	0.206	−0.137	−0.266, −0.007	0.038
Necrotic core volume	0.271	−0.013, 0.555	0.062	0.074	−0.055, 0.203	0.261

ACS, acute coronary syndrome; CI, confidence interval; SMD, standardized mean difference

### Meta-regression

**Fig. 7:**
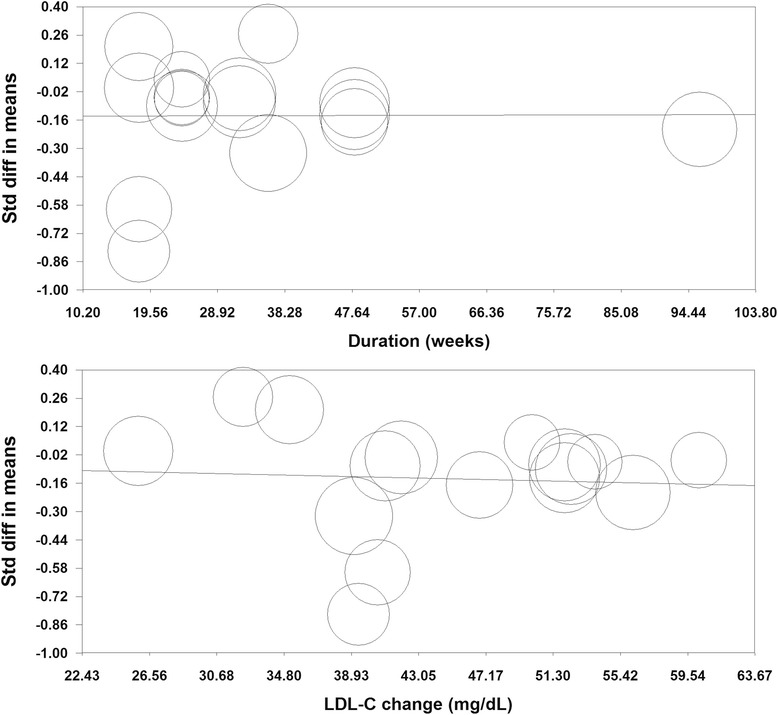
Random effects meta-regression plots of the association between mean changes in plaque volume with treatment duration, and changes in plasma low-density lipoprotein cholesterol (LDL-C) concentrations. The size of each circle is inversely proportional to the variance of change. Meta-regression was performed using unrestricted maximum likelihood method

Meta-regression analysis was conducted to assess the association between statin-induced changes in PV with duration of statin therapy and respective changes in plasma LDL-C concentrations as potential confounders. In meta-regression analysis, the impact of statins on PV was found to be independent of treatment duration (slope: 0.00007; 95 % CI: −0.006, +0.006; *P* = 0.980). Likewise, statin-induced reduction in PV was not found to be significantly associated with LDL-C reductions (slope: −0.002; 95 % CI: −0.015, +0.011; *P* = 0.788) (Fig. 7). Further analyses did not reveal any significant association between statin-induced changes in PV and other potential confounders including age, dose (atorvastatin), age, proportion of males, proportion of diabetics, proportion of smokers and baseline LDL-C (Table 4).

**Table 4:** Impact of potential confounders on changes in plaque volume following statin therapy in random-effects meta-regression

Confounder	Slope	95 % CI	*P* value
Age (years)	0.009	−0.020, 0.039	0.537
% Males	−0.011	−0.024, 0.002	0.106
% Diabetics	0.003	−0.002, 0.008	0.255
% Smokers	−0.004	−0.009, 0.0004	0.075
Dose (mg/day)^a^	−0.007	−0.015, 0.001	0.091
Baseline LDL-C (mg/dL)	0.004	−0.007, 0.016	0.435

^a^Restricted to atorvastatin trials. CI, confidence interval; LDL-C, low-density lipoprotein cholesterol

### Publication bias

**Fig. 8:**
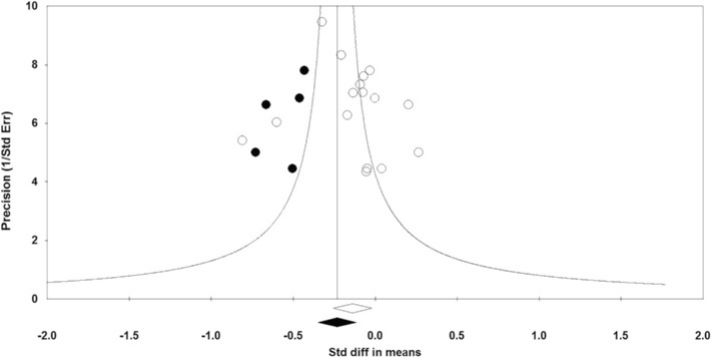
Funnel plot detailing publication bias in the studies reporting the impact of statin therapy on plaque volume. Open circles represent observed published studies; closed circles represent imputed unpublished studies

The results of Egger’s linear regression (intercept = 0.860, standard error = 1.866; 95 % CI: −3.142, +4.861, *t* = 0.461, df = 14.00; two-tailed *P* = 0.652) and Begg’s rank correlation (Kendall’s tau with continuity correction = 0.025, *Z* = 0.135; two-tailed *P* = 0.893) tests did not provide any proof of significant publication bias for the decreasing effect of statin therapy on PV. However, the funnel plot of precision (1/standard error) by effect size (SMD) was found to be asymmetric and suggestive of potential publication bias. The observed publication bias was imputed using trim-and-fill correction. This correction suggested no asymmetry on the right of the mean, while five potentially missing studies were imputed on the left of the mean leading to a corrected effect size that was significant: SMD: −0.232 (95 % CI: −0.351, −0.114). The ‘fail-safe N’ method indicated that 38 theoretically missing studies would need to be added to the analysis before the overall effect size becomes trivial. Funnel plot of the impact of statins on plaque volume is illustrated in Fig. 8.

## Discussion

The present systematic review and meta-analysis provides a comprehensive assessment of the impact of statin therapy on coronary plaque composition assessed with VH-IVUS. We observed a significant effect of statin therapy on plaque volume (however with no significant changes in lumen volume), external elastic membrane, fibrous and dense calcium volumes, while fibro-fatty and necrotic core tissue volumes remained statistically unchanged.

The potential reason for obtaining these results may lie in the fact that foam cells function as a substrate for the progression of necrosis [31]. The existence of foam cells and non-load-bearing lipid pools enzymatic together with destruction of collagen by matrix metalloproteinases, and microcalcifications might produce a TCFA, increasing the risk of plaque rupture and MACE [32]. However, statins have been associated with increase in fibrous cap thickness in optical coherence tomography (OCT) studies [33]. In these OCT studies, only assessment of the near field can be achieved due to the poor penetration of the technology and therefore the quantification of fibrous tissue in the total plaque cannot be obtained. In our meta-analysis that included only VH-IVUS studies, we observed a global decrease in fibrous tissue associated with statin treatment. In other words, there may be two differential effects of statin treatment, on the one hand a focal increase in cap thickness and on the other hand a global decrease in fibrous tissue. This hypothesis needs further investigation.

Increased quantities of calcium in coronary plaques have been linked to negative remodeling [34, 35], in contrast to increased lipid and fibro-fatty elements usually seen in positively remodeled lesions [36, 37]. Moreover, ACS and histological features of plaque vulnerability such as a large lipid core and high macrophage content seems to be associated with a positive coronary arterial remodeling [38].

Many studies such as the Myocardial Ischemia Reduction with Aggressive Cholesterol Lowering (MIRACL) [39] and the Pravastatin or Atorvastatin Evaluation and Infection Therapy–Thrombolysis in Myocardial Infarction 22 (PROVE IT-TIMI 22) [40] have reported that intensive statin therapy reduces MACE in patients with coronary heart disease. Significant plaque burden, extensive remodeling and calcification have been regarded as fundamental morphologies of high-risk plaques leading to MACE [41]. It has been shown that statin therapy improves plaque hyperechogenicity without a considerable decrease in plaque volume, suggesting that statins might influence coronary artery plaque composition [42]. Moreover, in non-culprit, high-risk coronary lesions after the onset of ACS, statins proved to be beneficial for regression and stabilization of vulnerable plaques [41]. However, the effect of statin therapy on plaque volume and composition might essentially differ by statin preparations, doses, duration of therapy, methods of imaging, as well as plaque localization. In the Reversal of Atherosclerosis with Aggressive Lipid Lowering (REVERSAL) trial [43], moderate lipid-lowering therapy with 40 mg of pravastatin did not stop plaque progression, while treatment with 80 mg of atorvastatin did. The first study showing reduction on plaque size was the a Study to Evaluate the Effect of Rosuvastatin on Intravascular Ultrasound-Derived Coronary Atheroma Burden (ASTEROID) trial with 40 mg of rosuvastatin [44]. However, these trials have only evaluated quantitative changes of coronary artery plaque using gray-scale IVUS and did not study plaque composition changes. Our meta-analysis showed that statin therapy reduces atheroma plaque volume, however with no significant changes in lumen volume. It also influences plaque composition reducing fibrous volume, however with no significant changes in fibro-fatty and necrotic core tissue volumes. Although these results differed between available studies [14, 21–28], these observations confirm the changes in plaque composition affecting lesion size and plaque stability (changes the composition of plaques from fatty to fibrous). On the other hand, the lack of effect on necrotic material is highly concerning for the field, given that the outcome studies in this field have largely supported the findings that TCFA is associated with adverse outcomes [45].

Statin therapy induced a significant regression of IVUS-measured coronary plaque volume, especially when reaching the target LDL-C level, as shown in a meta-analysis of gray-scale IVUS studies investigating temporal modifications in coronary plaque volume [46]. However, conventional gray-scale IVUS compared with VH-IVUS method has many limitations in the evaluation of atheromatous plaque composition and identification of a vulnerable plaque prior to rupture [47–49]. Another study indicated that VH-IVUS may potentially allow the best detection of features associated with future plaque rupture, increasing the probability of superior risk stratification at the moment of percutaneous coronary intervention [50].

The present meta-analysis has several limitations. Most importantly, there were few eligible prospective trials, and most had small numbers of patients. Furthermore, the included studies were heterogeneous regarding factors such as population characteristics (different statins, doses and duration of treatment), study design and VH-IVUS methodology (for example, in some of the included studies VH-IVUS was not performed in all patients and there were different IVUS catheters used in the included studies). There were only two studies controlled with placebo, and others compared high-intensity versus moderate/low-intensity statin therapy. Furthermore, VH-IVUS was only performed in one coronary vessel, which might not reflect changes in plaque features sampled from other regions of the coronary tree. Plaque volume might be also very variable when measured in mm^3^ across studies. Finally, the use of serial VH-IVUS imaging might be problematic, as it is ECG gated, so there is limited ability to precisely match segments.

## Conclusions

In conclusion, this meta-analysis of nine prospective studies comprising 16 statin-treated arms indicates a significant effect of statin therapy on plaque, external elastic membrane, fibrous and dense calcium volumes, while fibro-fatty and necrotic core tissue volumes remained statistically unchanged. Further large-scale, well-designed head-to-head trials are warranted to fully address the differential effects on these parameters with different statins.

## Abbreviations

ACC: American College of Cardiology
ACS: Acute coronary syndrome
AHA: American Heart Association
ASTEROID: A Study to Evaluate the Effect of Rosuvastatin on Intravascular Ultrasound-Derived Coronary Atheroma Burden
ATHEROREMO-IVUS: European Collaborative Project on Inflammation and Vascular Wall Remodeling in Atherosclerosis
BMI: Body mass index
CI: Confidence interval
CMA: Comprehensive Meta-Analysis
CVD: Cardiovascular disease
DBP: Diastolic blood pressure
EEMV: External elastic membrane volume
HDL-C: High-density lipoprotein cholesterol
hs-CRP: high-sensitivity C-reactive protein
LDL-C: Low-density lipoprotein cholesterol
LV: Lumen volume
MACE: Major adverse cardiovascular event
MeSH: Medical Subject Headings
MIRACL: Myocardial Ischemia Reduction with Aggressive Cholesterol Lowering
OCT: Optical coherence tomography
PRISMA: Preferred Reporting Items for Systematic Reviews and Meta-Analysis
PROSPECT: Providing Regional Observations to Study Predictors of Events in the Coronary Tree
PROVE IT-TIMI 22: Pravastatin or Atorvastatin Evaluation and Infection Therapy–Thrombolysis in Myocardial Infarction 22
PV: Plaque volume
REVERSAL: Reversal of Atherosclerosis with Aggressive Lipid Lowering
SBP: Systolic blood pressure
SD: Standard deviation
SEM: Standard error of the mean
SMD: Standardized mean difference
TC: Total cholesterol
TCFA: Thin-cap fibroatheroma
TG: Triglyceride
VH-IVUS: Virtual histology intravascular ultrasound
VIVA: VH-IVUS in Vulnerable Atherosclerosis
